# Efficacy of auriculotherapy in the control of pain, edema, and trismus following surgical extraction of the lower third molars: a split-mouth, randomized, placebo-controlled, and triple-blind study

**DOI:** 10.1007/s10006-023-01140-y

**Published:** 2023-02-03

**Authors:** Luigi Angelo Vaira, Andrea Massaiu, Giuseppe Massaiu, Giovanni Salzano, Fabio Maglitto, Jerome R. Lechien, Andrea Biglio, Giulio Visaloco, Pasquale Piombino, Federico Biglioli, Giacomo De Riu

**Affiliations:** 1 Maxillofacial Surgery Operative Unit, Department of Medicine, Surgery and Pharmacy, University of Sassari, Sassari, Italy; 2 Biomedical Science Department, PhD School of Biomedical Science, University of Sassari, Viale San Pietro 43B, Sassari, Italy; 3 Studio Massaiu, Sassari, Italy; 4 Department of Maxillofacial Surgery, University of Naples “Federico II”, Naples, Italy; 5 Department of Anatomy and Experimental Oncology, Mons School of Medicine, UMONS Research Institute for Health Sciences and Technology, University of Mons (UMons), Mons, Belgium; 6 Department of Otolaryngology-Head Neck Surgery, Polyclinic of Poitiers, Elsan Hospital, Poitiers, France; 7 Maxillofacial Surgery Department, San Paolo Hospital, ASST Santi Paolo E Carlo, University of Milan, Milan, Italy; 8 Dental School, University Hospital of Sassari, Sassari, Italy

**Keywords:** Acupuncture, Auriculotherapy, Third molar, Tooth extraction, Oral surgery, Pain, Edema, Trismus

## Abstract

**Background:**

The aim of this split-mouth, randomized, placebo-controlled, and triple-blind study was to evaluate whether auriculotherapy had any effect on the post-operative course after the extraction of third molars in terms of the control of pain, edema, and trismus.

**Materials and methods:**

The study included 42 patients (84 teeth) who had undergone a surgical extraction of the lower third molars. In each patient, the two extractions were randomly assigned to two study groups. In the therapy group, the patients underwent auriculotherapy with vaccaria seeds applied with patches in 6 ear points. In the control group, the patches were applied, without seeds, to the same ear points. After the extraction, the patients were asked to stimulate the ear points three times a day and whenever they felt pain. The patients were asked to keep a diary in which they assessed their pain by means of the Visual Analog Scale (VAS) for 8 days. Edema and trismus were assessed 1, 2, 3, and 8 days after surgery.

**Results:**

The differences between the two groups were statistically significant at the 12-h control (auriculotherapy group (AG) VAS 5.5 [IQR 4.25–6.75], placebo group (PG) VAS 6 [IQR 5–8], *p* = 0.040), after 24 h (AG VAS 5 [IQR 4–6], PG VAS 6 [IQR 4.25–7], *p* = 0.024), after 2 days (AG VAS 4 [IQR 3–5], PG VAS 4.5 [IQR 4–6], *p* = 0.044), and after 3 days (AG VAS 3 [IQR 0–5], PG VAS 4 [IQR 3–5], *p* = 0.024). Throughout the observation period, the AG took a significantly lower number of painkillers than the PG (AG 6 [IQR 4.25–7]; PG 8 [IQR 8–9], *p* < 0.001). There were no significant differences in the levels of edema and trismus between the two groups throughout the observation period.

**Conclusions:**

On the basis of the results of the present study, auriculotherapy can be considered as a cost-effective adjuvant pain reliever treatment in patients undergoing an extraction of the lower third molars.

## Introduction

Third molar extraction is one of the most commonly performed procedures in oral surgery. Pain, edema, and trismus represent the most common sequelae of this surgery and, even if only temporarily, significantly reduce the quality of life of these patients [1, 2]. Several therapies have been proposed to reduce the severity of these complications [3], including non-steroidal anti-inflammatory drugs (NSAID), acetaminophen, opioids, corticosteroids, antibiotics, and cryotherapy. In particular, NSAID, paracetamol, and corticosteroids are the most commonly used therapies for the control of pain, edema, and post-operative trismus and numerous therapeutic protocols have been proposed over time [4–7]. However, these drugs are burdened with various side effects, which can be frequent in the case of the high-dose therapies which are often required after surgical extraction of the third molars [8–10]. For this reason, it would be advisable to identify adjuvant therapeutic aids which could enable a reduction in the doses and duration of analgesic and anti-inflammatory therapies.

In recent years, increasing attention has been focused on alternative therapies such as acupuncture, herbal medicine, and homeopathy. Auriculotherapy is a branch of acupuncture which consists in treating diseases of the organism by stimulating specific points located in the auricle. It is based on anatomical maps that identify, on each ear, the projections of all the organs and functions of the human body. These points can be stimulated with different tools: seeds, needles, or laser or electric stimulation [11, 12]. Auriculotherapy has been shown to be effective as an adjuvant to normal analgesics in the treatment of the acute and chronic pain related to various diseases or experienced after surgery [13–15]. In relation to pain control in oral surgery, there are few trials involving acupuncture. A split-mouth, randomized, triple-blind study of 15 patients by Armond et al. [16] reported a significant reduction in edema levels in an auricular group without any effect on pain, trismus, and anxiety. On the contrary, Lao et al. [17] in their randomized, double-blind, placebo-controlled trial, while not finding any significant differences in pain levels between the two groups, discovered that subjects in the treatment group required significantly fewer analgesics. To the best of our knowledge, only one study has evaluated the efficacy of auriculotherapy in oral surgery in the control of inflammatory sequelae. In their split-mouth, randomized, single-blind study of 84 patients, Sampaio-Filho et al. [18] found no significant differences between the two groups in relation to any of the parameters analyzed in a study in which they stimulated the ear points with low-laser therapy. However, with this technique, as with “systemic” acupuncture, it is necessary that the stimuli be applied by an operator, rendering any routine application complicated in the sense that numerous appointments are required for each patient. One of the advantages of auriculotherapy is that the ear points can be stimulated directly by the patient through vaccaria seeds (Vaccaria Hispanica) applied to the auricle using patches. In this way, the patient him/herself can perform the stimulus without the need for any further evaluation by an operator.

Adopting this stimulation modality, Dellovo et al. [19], in their randomized split-mouth study on 30 patients undergoing extraction of the lower third molars, reported that auriculotherapy demonstrated an anxiolytic effect equivalent to midazolam, without any benzodiazepine-related side effects. However, to the best of our knowledge, there are no studies on the effect of auriculotherapy with this type of stimulation on the control of inflammatory sequelae after third molar extraction. The aim of this study has been to evaluate whether auriculotherapy has any effect on the post-operative course after the extraction of third molars in terms of the control of pain, edema, and trismus.

## Materials and methods

This split-mouth, randomized, placebo-controlled, and triple-blind study was conducted at the Department of Maxillofacial Surgery of the University Hospital of Sassari from 1st April 2021 to 15th March 2022. The study was approved by the ethics committee of the University Hospital of Cagliari (protocol no. PG/2021/7117) and was carried out in accordance with the guidelines of the consolidated standards of reporting trials (CONSORT) and the ethical principles of the Declaration of Helsinki.

The study included adult patients (> 18 years of age) who had undergone a surgical extraction of both lower third molars for orthodontic reasons. To be included in the study, both teeth in each subject had to have the same classification according to Pell and Gregory [20] and Winter [21]. Patients were excluded from the study if they met one of the following exclusion criteria:

- The presence of pericoronaritis or associated bone lesions;
- Acute or chronic pain present before surgery, even if in other anatomical sites and for other causes;
- Antibiotic, analgesic, or corticosteroid therapy in progress since before the surgery for other reasons;
- A severe systemic, psychiatric, or neurological disease;
- A difference of more than 5 min between the duration of the two extractions;
- Intra- or post-operative complications (hemorrhage, infection, alveolitis, dehiscence of the surgical wound, or deficit affecting the third branch of the trigeminal nerve);
- Previous malignancy, deformity, trauma, or surgery of the auricle;
- Any allergy or contraindication to taking NSAIDs.

Patients who met the inclusion and exclusion criteria and who agreed to participate in the study were then scheduled for two surgeries, 30 days apart, for the extraction of the two lower third molars.

The procedures were then randomized using an alpha-numeric spreadsheet that assigned each extraction for each patient to the two study groups:

- The auriculotherapy group (AG) or
- The placebo group (PG)

### Surgical procedure

All the extractions were performed by the same surgeon, with many years of experience in this procedure. The surgeon did not know the study group allocation. The surgery was performed on an outpatient basis under local anesthesia. After the elevation of a muco-periosteal flap, if necessary, the excess of the mandibular bone that covered the dental element was removed with high-speed rotary instruments. If appropriate, an odontotomy was performed to facilitate the tooth extraction. The surgical wound was closed by primary intention and the suture was removed after 8 days.

### Pharmacological therapy

All the patients underwent antibiotic prophylaxis with amoxicillin + clavulanic acid, 2 g 1 h before surgery. If the patient was allergic to penicillin, the prophylaxis was carried out with clindamycin 600 mg 1 h before surgery.

After the surgery, all patients were prescribed rest for 48–72 h, cryotherapy for the first 7 h, and an accurate oral hygiene with the use of topical gel based on chlorhexidine. In accordance with routine practice at our center, the patient was required to take ketoprofen lysine salts 80 mg, 1 sachet in case of pain up to a maximum of 3 sachets per day with a minimum interval of 8 h between sachets.

### The auriculotherapy group

**Fig. 1:**
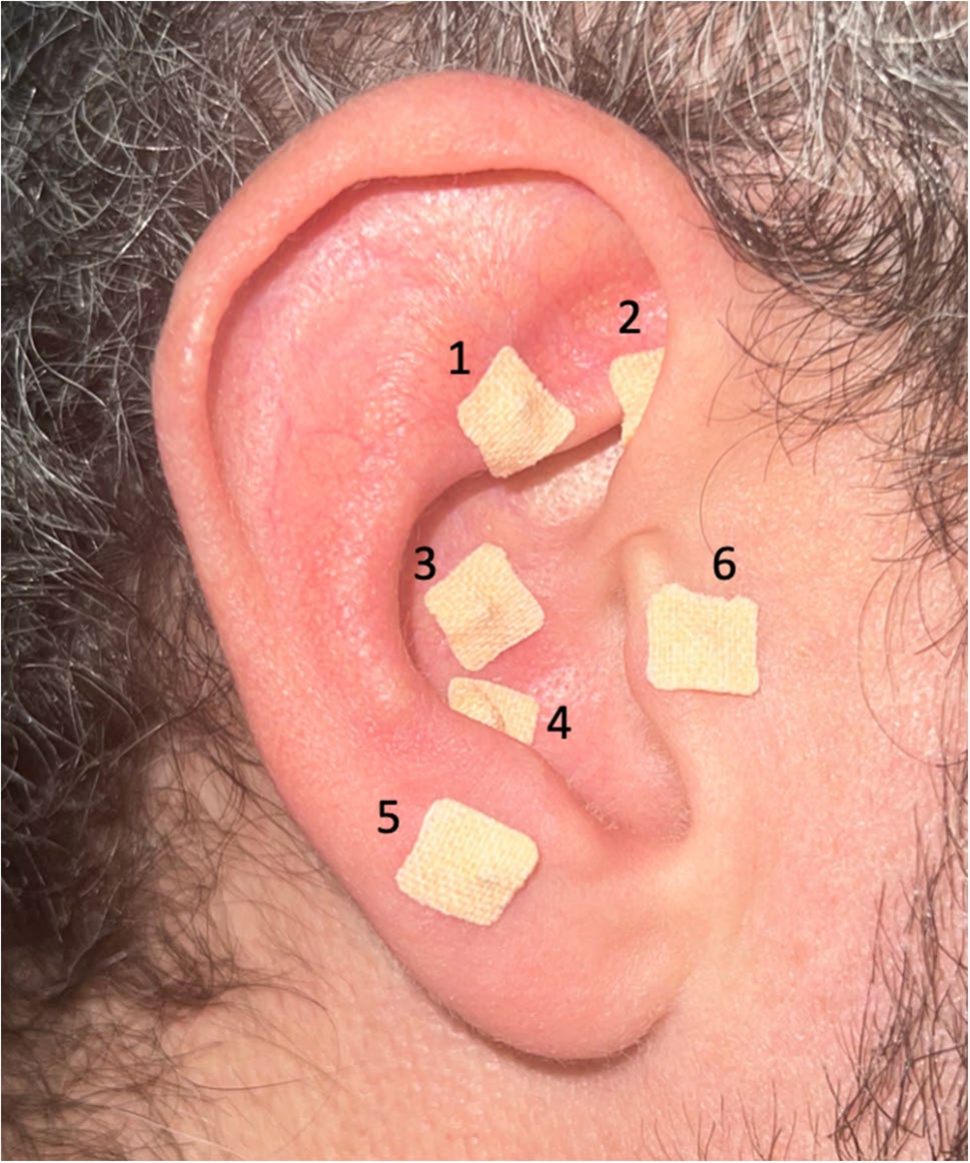
Auricular acupuncture points: (1) Shen Men, (2) Sympathetic (SNV), (3) Stomach, (4) Toothache 3, (5) Jaw, and (6) Adrenal

At the end of the surgery, an operator other than the surgeon who had performed the surgery applied the vaccaria seeds (Vaccaria Hispanica) using small patches (Dragon Acupuncture, EasyTech Trading Pte. Ltd., Singapore) on the following points of the auricle homolateral to the extraction (Fig. 1) [18, 22, 23]:

1. Shen Men: relieves pain, anxiety, and inflammatory diseases;
2. Sympathetic: activation of the sympathetic or parasympathetic nervous system with a reduction of any autonomic imbalance;
3. Stomach: reduces pain in the teeth;
4. Toothache 3: reduces pain in the lower teeth;
5. Jaw: reduces pain in the lower teeth, tension, and anxiety;
6. Adrenal (suprarenal): stimulates the production of hormones by the adrenal glands with a reduction of stress.

The patients were asked to massage each of the six points four times a day for 10 s. Moreover, they had to perform stimulation when they felt the onset of pain. The maneuver could be repeated if it led to an alleviation of symptoms. Otherwise, the patient was advised to take pain-relieving therapy as needed. The seeds were maintained in position until the suture was removed, 8 days after the surgery.

### The control group

At the end of the surgery, the same patches were applied to the same six points as for the patients in the AG, but without the vaccaria seeds. As in the AG, the patient was asked to alternately massage the points as soon as he/she felt the onset of pain and to take painkiller therapy if the maneuver was ineffective.

### Clinical parameters evaluated

Three clinical parameters were analyzed for each patient: pain, edema, and mouth opening.

All the measurements were carried out by the same researcher who was not aware of the allocation of the patients. The duration of the operation was also measured for each patient in order to establish whether there were significant differences between the two groups of procedures.

#### Post-operative pain

The patient was given an evaluation form asking him/her to report the pain present, by means of the VAS, assigning a score from 0 (no pain) to 10 (intolerable pain). The measurement took place 6, 12, and 24 h after the surgery and then at 24-h intervals until the stitches were removed. The patient was also asked to log each pain reliever taken during the observation period.

#### Post-operative edema

**Fig. 2:**
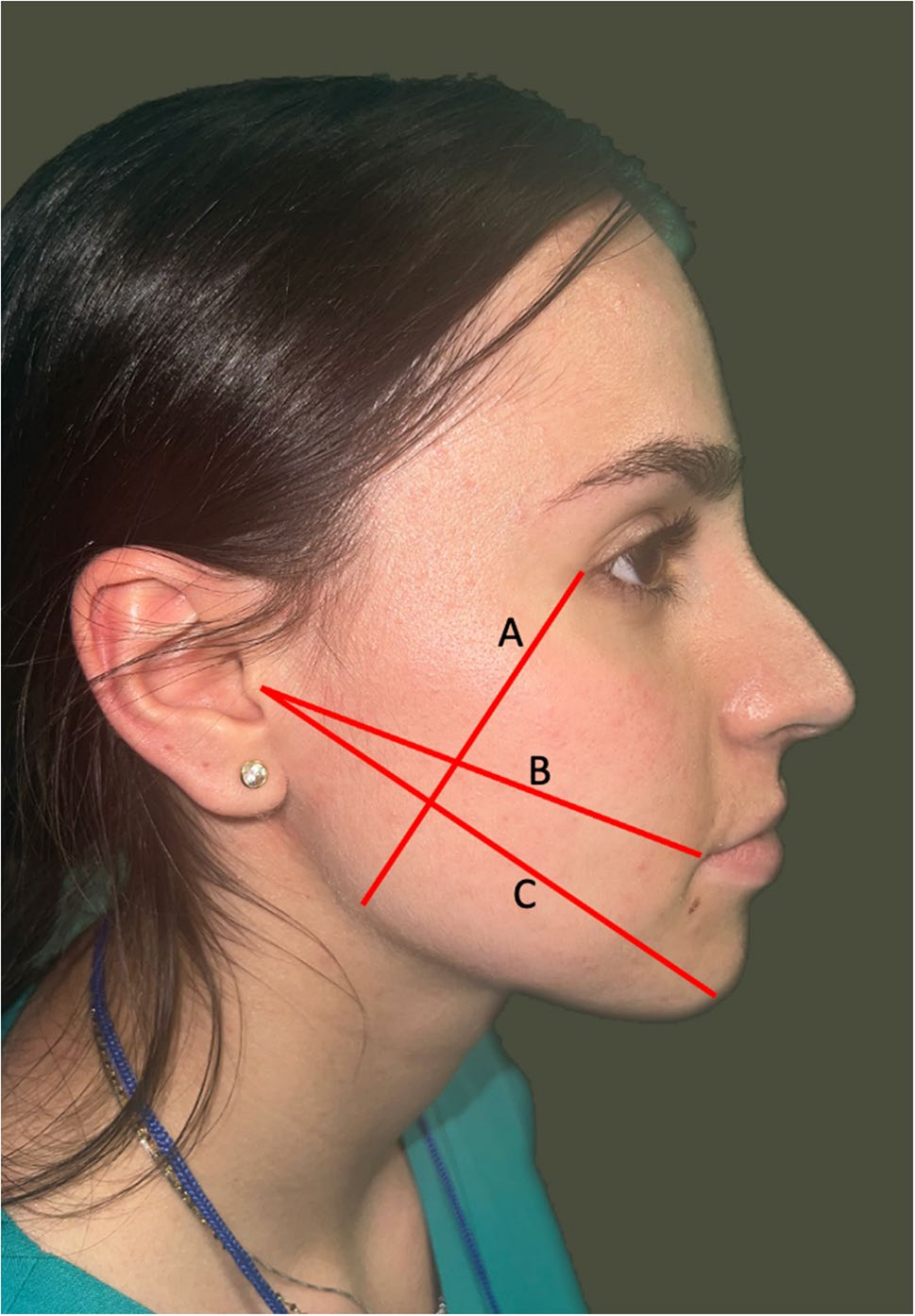
Edema assessment methodology. Line A: from the external canthus to the mandibular angle; line B: from the tragus to the labial commissure; and line C: from the tragus to the pogonion

The measurements were taken before the surgery (the baseline) and then after 24, 48, and 72 h and 8 days after the surgery. The sum of the three pre-operative measurements was considered the normality standard for each side (Fig. 2) [24]. The evaluation of the edema was quantified by subtracting the sum of the pre-operative measurements from the sum of the three post-operative measurements at each observation point.

#### Post-operative mouth opening

Mouth opening was used to assess the severity of any post-operative trismus. For the purpose of its determination, the distance between the occlusal margin of the upper and lower left central incisor was measured with a caliper. The measurement was carried out before the surgery (the baseline) and then after 24, 48, and 72 h and 8 days after the surgery. The difference between the pre-operative and post-operative measurements determined the severity of the trismus at each observation point.

### Statistical analysis

The calculation of the sample size was performed with G*power 3.1 (Heinrich Heine University Dusseldorf, Dusseldorf, Germany). The data used for the sample size calculation were based on the study by Sampaio et al. [18]: 95% power, 5% margin of error, and Cohen’s *D* 0.51. A minimum number of 42 patients (84 procedures) was thus determined.

The statistical analysis was carried out using SPSS 26.0 (IBM, Armonk, NY, USA). The variables are reported as an absolute number and as a percentage of the total. Descriptive statistics for the quantitative variables are reported as means ± the standard deviation or median [interquartile range — IQR].

The differences within each group for the variables which presented a pre-operative measurement were evaluated using the Wilcoxon test. This test was also used to analyze the differences between the two study groups for all the variables considered. The level of statistical significance was set at *p* < 0.05 with a confidence interval of 95%.

## Results

**Fig. 3:**
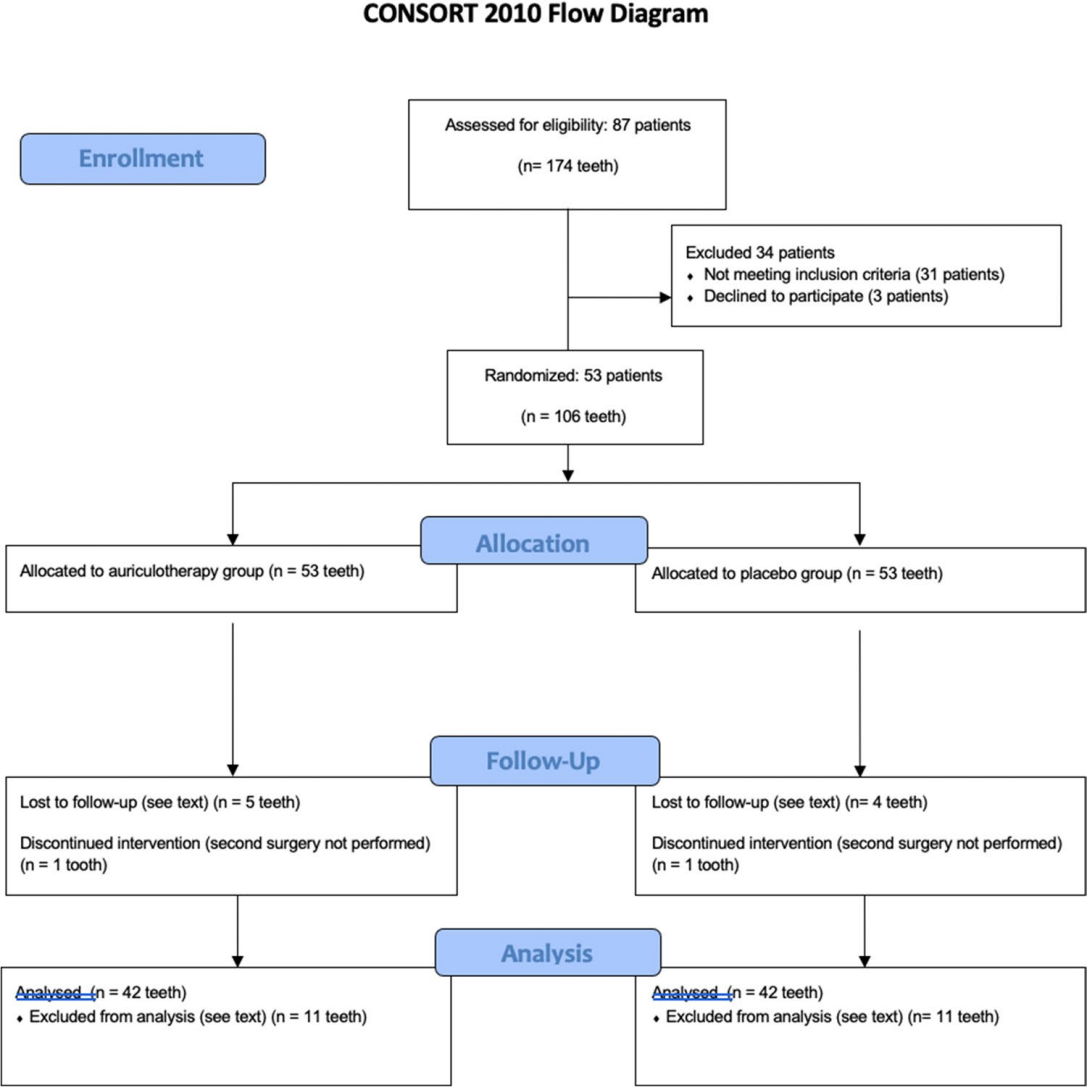
CONSORT flow diagram

Fifty-three patients who met the inclusion and exclusion criteria were included in the study (Fig. 3). Eleven patients were subsequently excluded for the following reasons: refusing the second surgery (2 cases); post-operative infection (1 case); temporary anesthesia of the lingual nerve (1 case); surgical wound dehiscence (1 case); alveolitis (2 cases); a significantly different duration of the two operations (1 case); and patients lost during follow-up or due to errors in completing the post-operative diary (3 cases).

Forty-two patients (84 procedures) were then considered for the analysis of the clinical parameters. The study group included 28 women and 14 men with an average age of 24.5 ± 6 years. The two study groups each included 42 procedures. The differences in the duration of the operations between the two groups were statistically insignificant (AG 17 [IQR 15–25], PG 17.5 [IQR 14.25–21; *p* = 0.659]). The extraction assigned to the AG was the first in 45.2% of cases and the second in 54.8%.

### Pain assessment results

**Fig. 4:**
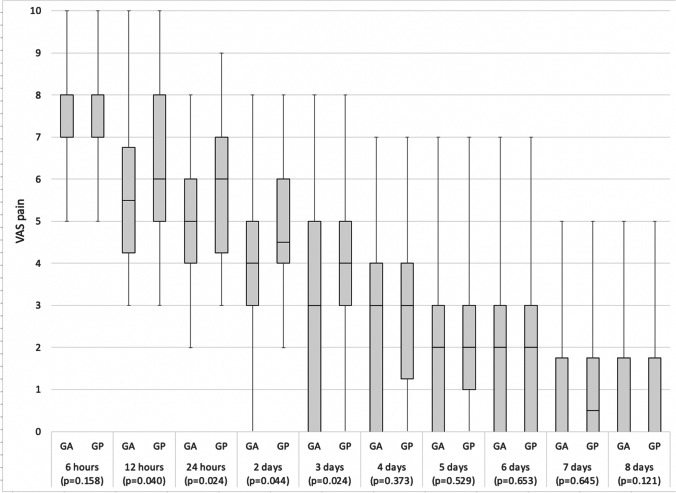
Comparison of the pain VAS scores between the two study groups during the observation period. Legend: GA: Auriculotherapy group; GP: Placebo group

The results of the pain assessment are shown in Fig. 4 and Table 1. The apex of pain detected by the patients was found at 6 h (AG VAS 7 [IQR 7–8], PG VAS 8 [IQR7–8], *p* = 0.158). The differences between the two groups were statistically significant at the 12-h control (AG VAS 5.5 [IQR 4.25–6.75], PG VAS 6 [IQR 5–8], *p* = 0.040), at 24 h (AG VAS 5 [IQR 4–6], PG VAS 6 [IQR 4.25–7], *p* = 0.024), at 2 days (AG VAS 4 [IQR 3–5], PG VAS 4.5 [IQR 4–6], *p* = 0.044), and at 3 days (AG VAS 3 [IQR 0–5], PG VAS 4 [IQR 3–5], *p* = 0.024). The differences between the two groups in the remainder of the observation period were not significant (Fig. 4) (Table 1).

**Table 1:** Results of the post-operative pain assessment

Observation time	Auriculotherapy group Median [IQR]	Placebo group Median [IQR]	Wilcoxon test *p*-value
6 h	7 [7, 8]	8 [7, 8]	0.158
12 h	5.5 [4.25–6.75]	6 [5–8]	0.040
24 h	5 [4–6]	6 [4.25–7]	0.024
2 days	4 [3–5]	4.5 [4–6]	0.044
3 days	3 [0–5]	4 [3–5]	0.024
4 days	3 [0–4]	3 [1.25–4]	0.373
5 days	2 [0–3]	2 [1–3]	0.529
6 days	2 [0–3]	2 [0–3]	0.653
7 days	0 [0–1.75]	0.5 [0–1.75]	0.645
8 days	0 [0–0.75]	0 [0–1.75]	0.121
No. of painkillers	6 [4.25–7]	8 [8, 9]	< 0.001

Overall, the AG patients took, throughout the observation period, a significantly lower number of painkillers than those in the PG (AG 6 [IQR 4.25–7]; PG 8 [IQR 8–9], *p* < 0.001).

### Edema assessment results

Table 2 provides a summary of the post-operative edema assessment in the two study groups. At all times of observation, the differences between the two groups were not statistically significant.

**Table 2:** Results of the evaluation of post-operative edema

Observation time	Auriculotherapy group Median [IQR]	Placebo group Median [IQR]	Wilcoxon test *p*-value
24 h	36 [31–42]	37 [31–45.75]	0.569
2 days	34.5 [30–38]	35 [31.25–38]	0.529
3 days	19 [16–21]	18.5 [16–21]	0.936
8 days	0 [0–4]	0 [0–4.75]	0.841

### Mouth opening assessment results

Table 3 provides a summary of the post-operative trismus assessment in the two study groups. At all times of observation, the differences between the two groups were not statistically significant.

**Table 3:** Results of the evaluation of post-operative trismus

Observation time	Auriculotherapy group Median [IQR]	Placebo group Median [IQR]	Wilcoxon test *p*-value
24 h	12 [10–16]	14 [10.25–15]	0.638
2 days	14 [12.25–16]	14 [11–16]	0.569
3 days	12 [10–16]	12 [10–14.75]	0.465
8 days	2 [0–5]	1 [0–5]	0.496

## Discussion

In the past, acupuncture has shown promising results in pain control following oral surgery [16, 17]. However, it often requires multiple sessions, engaging both the operator and the patient in numerous appointments, thereby reducing the cost/benefit ratio. In this sense, auriculotherapy can represent a simpler treatment, manageable directly by the patient for the entire duration of the post-operative period. However, its efficacy in the control of inflammatory symptoms in oral surgery and more specifically after the extraction of third molars is a largely unexplored topic. The only study [18] published so far involves stimulation of the ear points by low-level laser therapy with the implication of all the limiting factors reported for acupuncture.

The pain after extraction of the third molars is due to the surgical trauma induced to the soft and hard tissues and is generally proportional to the extent of the periosteal dissection [3]. The methodology of the present study was devised with the aim of minimizing the effect of any confounding factors (the experience of the surgeon, duration of the surgery, or difficulty of the extraction), and the split-mouth design allowed the use of each patient both as a case and as a control. Compared to the PG, the patients in the AG demonstrated lower pain levels throughout the observation period, with significant differences at 12 and 24 h and at 2 and 3 days. Moreover, the subjects of the AG needed to take significantly fewer NSAIDs during the post-operative period. These results contrast with those of other studies on acupuncture [16, 25–27] and auriculotherapy with low-laser stimulation which, although detecting lower levels of pain in the therapy groups, did not show significant differences with the placebo group. This discrepancy could lie in the main advantage of auriculotherapy by means of stimulation through seeds, namely that the stimulation of the auricular points can be performed when the pain arises and not at fixed and sporadic moments, as is the case with acupuncture or auriculotherapy with other types of stimulation.

Post-surgical edema is also related to iatrogenic trauma and tissue manipulation. The maximum level of edema is generally reached 48 h after surgery with a complete regression around the seventh day [3, 16]. Several previous studies have shown a certain level of efficacy of acupuncture in controlling edema by increasing cortisol levels [16, 28, 29]. The choice of applying a stimulation also on the “adrenal” auricular point was made precisely to induce an effect on the secretion of glucocorticoid hormones by the adrenal glands [23]. However, the differences between the two study groups were not significant throughout the observation period. This is in line with previous findings in relation to low-laser stimulation auriculotherapy [18]. In the future, it will be interesting to study the relationship between the stimulation of the adrenal auricular point and the levels of cortisol in the blood.

The limitation of the mouth opening following extraction of the third molar is related to pain and contracture of the chewing muscles [30]. Auriculotherapy could therefore reduce trismus by acting on both main causal factors [31]. However, the differences between the two groups were not significant in all the observation periods.

On the basis of these results, it is possible to hypothesize that auriculotherapy does not offer significant advantages on the local control of the inflammation underlying the edema. Conversely, pain is not only mediated by local inflammation but also by higher processes on which auricular therapy appears to have some effect. It is well known that acupuncture is able to produce a reduction in pain by acting on the pain control centers in the brain by inducing the release of endorphins and other neurohumoral factors (i.e., neuropeptide Y and serotonin), increasing the release of adenosine in the tissues and modulating the activity of the neocortical limbic and paralimbic network [32].

The split-mouth design of the study, the inclusion only of subjects who had teeth with the same radiological classification, and the exclusion of cases in which the two surgeries had significantly different durations allowed us to obtain highly reliable results. However, a possible limitation of the study is related to the difficulty in achieving patient blinding. The patients in the PG may in fact perceive that the seed is not present under the patch but only in this way was it possible to ensure that no stimulus could be applied to the auricular points. However, patches without a seed, placed in the same ear points, are commonly used as placebos and are considered a reliable sham control method [19, 24, 33].

## Conclusions

On the basis of the results of the present study, auriculotherapy has proven to be a safe and effective method for assisting analgesic drug therapy for pain control after the surgical extraction of lower third molars. The patients treated with auriculotherapy demonstrated significantly lower pain levels and had to take significantly fewer analgesics. In contrast, auriculotherapy had no significant effect on the control of edema and trismus. The stimulation of the auricular points by means of vaccaria seeds, compared to other methods, can be carried out directly by the patient and does not require frequent post-operative checks, which would limit the application on a large scale and might reduce patient compliance. For these reasons, auriculotherapy can be considered as a cost-effective adjuvant pain relief treatment in patients undergoing extraction of the lower third molars.

## Data Availability

The data that support the findings of this study are available on request from the corresponding author (LAV).
